# X chromosome-encoded microRNAs in immune regulation: sex differences and clinical implications

**DOI:** 10.3389/fgwh.2026.1758961

**Published:** 2026-02-19

**Authors:** Valeria Lodde, Valentina Margarita, Myriam Gorospe, Ilaria Campesi

**Affiliations:** 1 Department of Biomedical Sciences, University of Sassari, Sassari, Italy; 2 Laboratory of Genetics and Genomics, National Institute on Aging Intramural Research Program, National Institutes of Health, Baltimore, MD, United States

**Keywords:** autoimmune diseases, immune response, sex differences, vaccine responses, x-resident miRNAs

## Abstract

Sex-based differences in immune function influence susceptibility to infections and predisposition to autoimmunity, with women showing both stronger immune responses and a higher burden of autoimmune and chronic inflammatory diseases. While sex hormones contribute to these differences, accumulating evidence highlights a central role for the X chromosome, which is enriched in immune-related genes and subject to complex regulatory mechanisms such as X-chromosome inactivation, skewing, escape from inactivation, and imprinting. Within this context, X chromosome–encoded microRNAs (miRNAs) have emerged as key post-transcriptional regulators of immune homeostasis. The X chromosome harbors the highest density of miRNAs in the human genome, many of which target pathways involved in immune activation, tolerance, and tumorigenesis. Notably, some X-resident miRNAs escape X-chromosome inactivation, leading to female-biased expression that may enhance immune reactivity but also predispose to loss of tolerance and autoimmunity. In this minireview, we summarize current knowledge on X chromosome–encoded miRNAs in immune regulation, discuss how their sex-biased expression patterns may contribute to female predominance in autoimmune diseases, and explore their potential utility as biomarkers and therapeutic targets for sex-aware precision medicine in inflammatory, autoimmune disorders and vaccine responses.

## Introduction

Sex-based differences in immune function are among the most consistent and biologically significant forms of sexual dimorphism in humans, influencing both susceptibility to infectious diseases and predisposition to autoimmunity. Females generally exhibit stronger innate and adaptive immune responses compared to males, with enhanced antigen presentation, higher antibody titers following vaccination, and more robust activation of T and B lymphocytes (1). This heightened immune responsiveness provides superior protection against infectious agents but also contributes to a higher incidence and greater severity of autoimmune and chronic inflammatory diseases such as systemic lupus erythematosus (SLE), rheumatoid arthritis (RA), multiple sclerosis (MS), and autoimmune thyroiditis, which affect women up to eight times more frequently than men (2–4).

Although sex hormones, particularly estrogens, play a central role in modulating inflammatory processes and promoting their resolution, sexual dimorphism in immune function emerges as early as during fetal development (5). In this regard, accumulating evidence indicates that genetic and epigenetic mechanisms linked to the X chromosome play an equally critical role in determining immune dimorphism (6). The X chromosome carries the highest density of immune-related genes in the human genome, including *TLR7*, *TLR8*, *IL2Rγ*, *FOXP3*, *CXCR3*, and *CD40L*, which encode key regulators of immune activation, tolerance, and inflammatory signaling (7, 8). In females, the presence of two X chromosomes introduces additional layers of genetic and epigenetic complexity. To ensure dosage compensation of genes residing on the X chromosome, one X chromosome in females undergoes random inactivation (X-chromosome inactivation, XCI) during early embryogenesis (9). However, an estimated 15% of X-linked genes escape XCI, potentially contributing to sexual dimorphism in X-linked gene expression. In addition to XCI escape, other regulatory mechanisms, including skewed XCI, characterized by nonrandom silencing of the X chromosome, and parent-of-origin-specific imprinting of genes located on the X chromosome, further influence sex-dependent differences in X-linked gene expression (10).

Within this chromosomal landscape, a considerable number of microRNAs (miRNAs) located on the X chromosome have been identified as critical modulators of immune homeostasis, further reinforcing the concept of a female immunological advantage (11). MiRNAs are small non-coding RNAs spanning 21–24 nucleotides that regulate gene expression post-transcriptionally. MiRNAs are transcribed as primary transcripts (pri-miRNAs) and processed by the microprocessor complex composed of Drosha and DGCR8, exported to the cytoplasm via exportin 5 (XPO5)/RanGTP, and finally cleaved by Dicer into mature miRNA duplexes that integrate into the RNA-induced silencing complex (RISC); upon integration, miRNAs suppress target protein production by either degrading the target mRNAs and/or suppressing its translation (12–17).

Through their impact on protein expression patterns, miRNAs regulate a broad range of biological processes, including cell proliferation, differentiation, apoptosis, and immune signaling, and their dysregulation contributes to the pathogenesis of numerous diseases, including cancer and autoimmune disorders (18). Notably, comprehensive genomic distribution analyses have demonstrated that the X chromosome exhibits the highest density of miRNA sequences among all chromosomes (19), with approximately 10% of known human miRNAs residing on the X chromosome (20). According to the miRBase database (https://www.miRBase.org), 118 miRNAs are mapped to the X chromosome, and 62 of them are validated as high-confidence entries as (Supplementary Table 1). A substantial proportion of these X-residing miRNAs are involved in immune regulation and tumorigenesis, two biologically interconnected processes that share common molecular pathways as well as in other essential cellular pathways. Using *in silico* miRNA target prediction algorithms, the possible functional roles of miRNAs residing on the X chromosome were assessed by identifying the pathways implicating proteins encoded by their target mRNAs. Supplementary Table 2 summarizes the pathway analysis of X-resident miRNAs. From this analysis, 75 signaling pathways were identified, of which approximately 23% are immune-related and 28% are associated with tumorigenesis. Among the pathways identified, representative immune- and tumor-related pathways include proteoglycans in cancer*,* viral carcinogenesis, endocytosis, bacterial invasion of epithelial cells, renal cell carcinoma and TGF-beta signaling pathway. Importantly, some X chromosome-resident miRNAs appear to escape XCI, resulting in female-biased expression patterns that may amplify immune reactivity and influence the balance between protection and pathology (21). Although a comprehensive and high-resolution map of miRNAs escaping XCI is still lacking, and direct quantitative evidence remains limited, accumulating data support the notion that a subset of X-resident miRNAs may evade silencing (21). Notably, several miRNAs are embedded within host genes that have been demonstrated to escape XCI, suggesting that these miRNAs may share the same epigenetic status (21). Examples include miR-548am, hosted within the *CTPS2* gene, and miR-374 and miR-421, located within the long non-coding RNA *FTX*, both reported to escape XCI (22, 23).

In addition to genomic context, functional evidence also supports a role for specific X chromosome-resident miRNAs in XCI regulation (24). Lou et al. identified six miRNAs, miR-106a, miR-363, miR-340, miR-34b, miR-30e, and miR-181a, as regulators of XCI (24).

**Figure 1:**
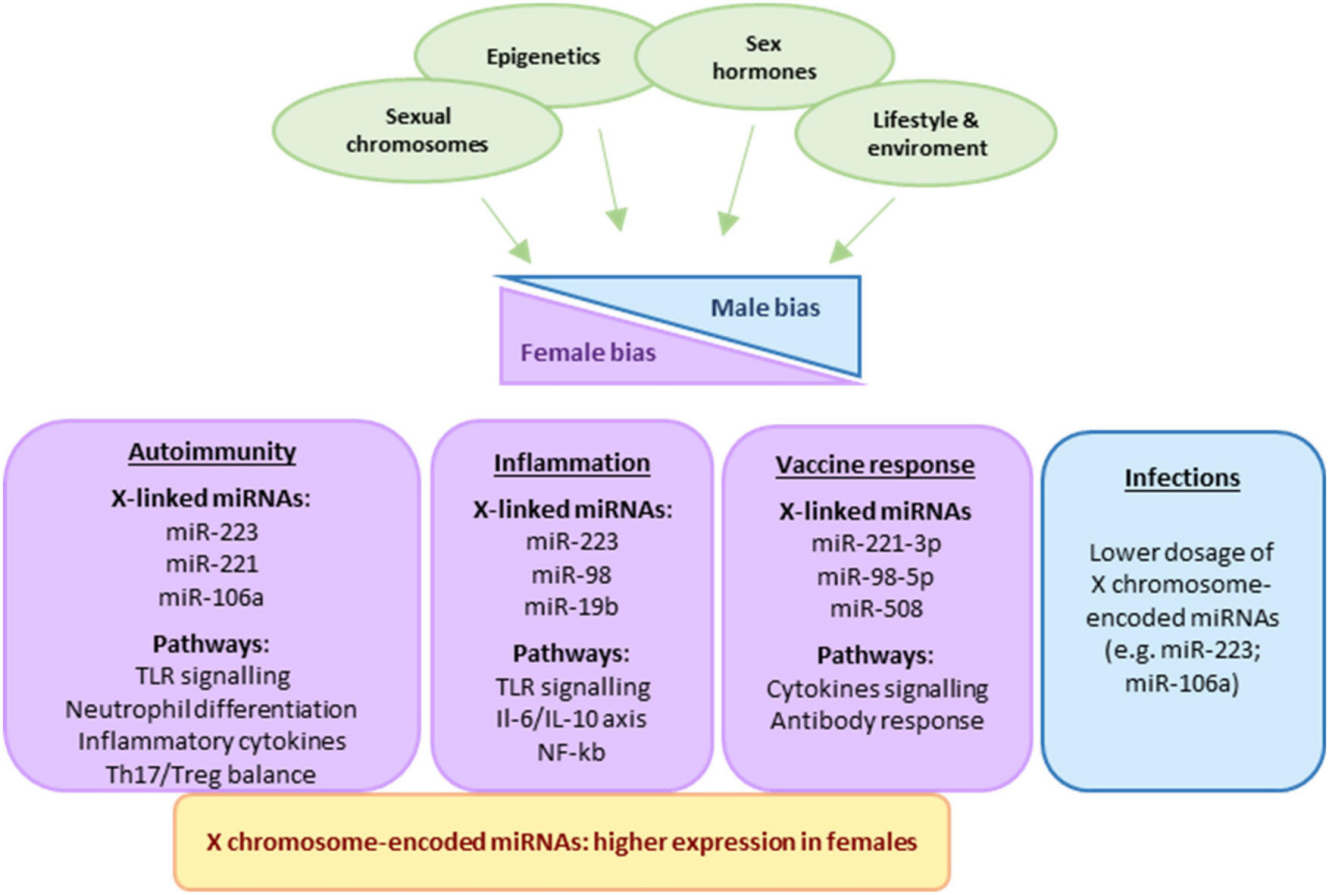
Sex differences in immune responses arise from the combined influence of sex hormones, sex chromosomes, epigenetic mechanisms, and environmental factors. X-chromosome–encoded miRNAs, which are more highly expressed in females, contribute to female-biased immune activation and differential susceptibility to autoimmunity, inflammation, vaccine responses, and infections.

Overexpression of specific X chromosome-encoded miRNAs can potentiate pro-inflammatory signaling cascades or impair immune tolerance mechanisms, thereby contributing to the female predominance observed in autoimmune diseases (3). The interplay among hormonal factors, immune-related genes, and X-encoded miRNAs thus represents a critical axis in the regulation of sex-dependent immune responses (Figure 1).

This minireview aims to provide a comprehensive overview of the emerging role of X chromosome-encoded miRNAs in the regulation of immune function, highlighting how their differential expression contributes to sex-based disparities in the incidence and severity of inflammatory and autoimmune diseases, and discussing their potential implications for the development of sex-specific diagnostic and therapeutic strategies.

### Search strategy

A comprehensive literature search was performed using MEDLINE/PubMed and Google Scholar as the primary databases, supplemented by manual searches of additional scientific sources and the reference lists of key meta-analyses and review articles to ensure completeness. The search strategy targeted the following thematic domains: sex differences in inflammation, sex differences in immune responses, X chromosome genes and inflammation, X chromosome genes and immunity, miRNAs and immunity, miRNAs and inflammation, and X chromosome genes and miRNAs, vaccines, vaccination. A combination of Medical Subject Headings (MeSH) and free-text keywords was employed, applying Boolean operators (“AND” and “OR”) to optimize the sensitivity and specificity of the search. All identified records were initially screened based on titles and abstracts to assess relevance. Full-text versions of potentially eligible articles were retrieved and evaluated for inclusion according to their methodological quality and pertinence to the review topic. No restrictions were applied regarding publication year, while only articles written in English were considered. The literature search was last updated in January 2026, and the list of X-chromosome-encoded miRNAs was identified using miRBase (https://www.miRBase.org). The miRNA pathway interactions were explored using the DIANA miRPath v.3 web-based computational tool (http://snf-515788.vm.okeanos.grnet.gr/) (25). This software performs enrichment analysis of miRNA targets associated with each set of miRNA target genes in relation to KEGG pathways. KEGG pathway enrichment analysis enables the investigation of biological pathways and molecular interactions linked to target genes.

### X chromosome-encoded miRNAs in immune regulation

**Figure 2:**
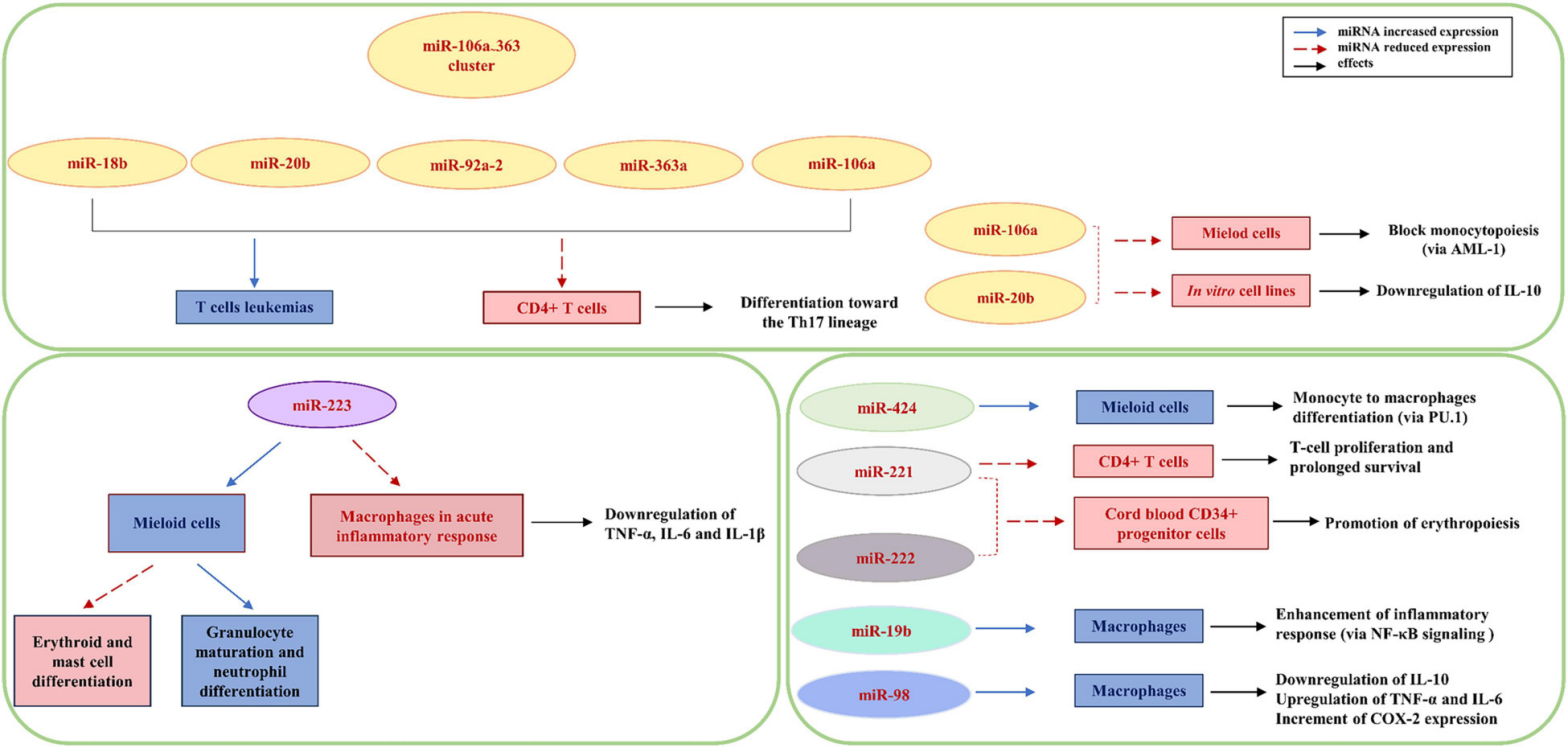
Role of X-linked miRNAs in immunity and inflammation. The figure summarizes the involvement of different miRNAs in the regulation of immune cell differentiation, and inflammatory responses. Solid blue arrows indicate miRNAs increased expression, while dashed red arrows indicate reduced expression of selected miRNAs.

Several X-linked miRNAs have been shown to exert critical functions in immunity and inflammation (Figure 2), potentially contributing to sex-dependent differences in immune responses as well as to variations in the dynamics and magnitude of the inflammatory reaction (11, 26).

Among the X chromosome-resident miRNAs implicated in immune regulation, miR-223 is one of the most extensively studied. It is predominantly expressed in cells of the granulocytic lineage, with expression levels progressively increasing during granulocyte maturation and decreasing during erythroid and mast cell differentiation (27, 28). Functionally, miR-223 acts as a key regulator of neutrophil differentiation from myeloid precursors and serves as a negative modulator of the inflammatory response, particularly by fine-tuning the acute inflammatory response following pathogen recognition through Toll-like receptors (TLRs) (29). Four additional X-linked miRNAs, miR-106a, miR-424, miR-542, and miR-503, are implicated in hematopoietic lineage differentiation (11). MiR-106a has been shown to negatively regulate monocytopoiesis by targeting AML-1, a key inducer of monocyte differentiation and maturation (30). MiR-106a belongs to the miR-106a∼363 cluster, which also includes miR-18b, miR-20b, miR-19b-2, miR-92a-2, and miR-363, all residing on the X chromosome and involved in fine-tuning T helper cell differentiation toward the Th17 lineage (31). Notably, overexpression of these miRNAs has been reported in human T-cell leukemias (32). Furthermore, the X-resident microRNAs miR-424, miR-503 and miR-222, promote monocytic differentiation (33). In particular, miR-424 regulates the myeloid-specific transcription factor SPI1 (PU.1) and promoting monocyte to macrophage differentiation by targeting NFI-A (Nuclear Factor I A) (33).

MicroRNAs miR-221 and miR-222 are downregulated during erythropoiesis, and this reduction may promote erythropoiesis by the regulation of key functional proteins (34). MiR-221 suppresses genes involved in T-cell proliferation and survival, while inhibition of miR-221 results in enhanced T-cell proliferation and prolonged survival, suggesting that miR-221 functions as a negative feedback regulator to maintain immune homeostasis (35). In addition, miR-106a and miR-20b have been shown to modulate the expression of the anti-inflammatory cytokine IL-10 (36, 37). Furthermore, miR-19b promotes nuclear factor kappa B (NF-*κ*B) signaling and is implicated in the enhancement of inflammatory responses (38). MiR-98 directly targets the 3′ untranslated region of IL-10 mRNA; accordingly, its overexpression suppresses TLR4-induced IL-10 production, enhancing COX-2 expression and increasing expression of pro-inflammatory cytokines, including TNF-α and IL-6, thereby amplifying the inflammatory response (39).

Taken together, these findings highlight the pivotal role of X chromosome-encoded miRNAs in regulating inflammatory pathways, myeloid cell differentiation, and innate immune responses. It is therefore plausible that dysregulation of these X-linked miRNAs contributes to the observed sex-based differences in susceptibility and severity of immune-mediated diseases.

### X chromosome-encoded miRNAs in autoimmunity

Autoimmunity diseases (ADs) comprise a group of disorders in which the immune system aberrantly targets self-antigens, leading to chronic inflammation and subsequent tissue damage (40). Common ADs include RA, SLE, MS, type 1 diabetes mellitus (T1D), psoriasis, Graves’ disease, inflammatory bowel disease, and Sjögren's syndrome. The majority of ADs show a marked female predominance, with female-to-male ratios ranging from 2:1 to 25:1 in certain conditions (41). However, not all ADs occur exclusively or preferentially in females (42). MiRNAs play a key role in the pathogenesis of several ADs (43–48). Altered miRNA levels are observed in most ADs and have negative effects on B and T cells differentiation, homeostasis and activation, and differentiation of dendritic cells and macrophages via Toll-like receptors (49–52).

#### Rheumatoid arthritis

The abnormal expression of X-encoded miRNAs in RA patients has been analyzed mainly in the context of T cell differentiation, production of inflammatory cytokines, and B cell activation (53, 54). Imbalances in miR-223 have been reported in the immune cells of RA patients (55). MiR-223 is intensely expressed in RA synovium, regulates the differentiation of osteoclasts, and has important roles in the state of RA joints (56). Furthermore, Khalifa et al. reported that the expression levels of five X-encoded miRNAs (miR-221, miR-222, miR-532, miR-106a, and miR-98) were significantly different between peripheral blood mononuclear cells (PBMCs) of RA patients and healthy controls when stratifying by sex, and the expression levels of four miRNAs (miR-222, miR-532, miR-98, and miR-92a) were significantly different between RA females and males (57).

Other miRNAs encoded in the X chromosome were found to have an important role in RA. MiR-19a/b regulates the expression of TLR2 in RA synovial fibroblasts (RASFs), which are key effector cells in RA pathogenesis and contribute significantly to joint inflammation and destruction (58). Through the downregulation of TLR2, miR-19a/b reduces inflammatory responses by decreasing the production of IL-6 and MMP-3 (58). The expression levels of miR-92a were markedly reduced in RA synovial tissue and RASFs, suggesting its potential involvement in RA pathogenesis (59, 60). Restoration of miR-92a levels suppresses the proliferation and migration of RASFs, suggesting an inhibitory role of this miRNA in RA progression (61). Mechanistically, miR-92a was shown to inhibit cell proliferation and migration through the downregulation of AKT2, a member of the serine/threonine kinase family that is involved in key cellular processes such as metabolism, survival, and proliferation. Collectively, the miR-92a/AKT2 signaling axis plays a critical role in the pathogenesis of RA and may represent a promising therapeutic target for disease prevention and treatment (61). MiR-188-5p has been shown to target cell migration-inducing and hyaluronan-binding protein (CEMIP) and to indirectly regulate the expression of collagen type I alpha genes (COL1A1 and COL12A1) in RASFs. Reduced levels of miR-188-5p lead to enhanced activation and migration of RASFs (62). MiR-221 and miR-222 play an important role in the pathogenesis of RA. Elatta et al. reported that the expression levels of miR-221 and miR-222 were significantly upregulated in patients with RA, showing a positive correlation with disease activity (63). In particular, the overexpression of miR-221 has been shown to enhance the production of pro-inflammatory cytokines, promote the activation and migration of RASFs, and confer resistance to apoptosis (64).

#### Systemic lupus erythematosus

Alterations in miRNAs expression have been observed in various immune cells of patients with SLE. The first analysis of differentially expressed miRNAs in SLE was conducted by Dai et al., who identified distinct expression patterns in several miRNAs in PBMCs from SLE patients compared to healthy controls (65). Over the years, multiple studies have confirmed that SLE patients exhibit unique miRNA expression profiles, including circulating miRNAs (66, 67). Several X-encoded miRNAs are dysregulated in SLE. Notably, lower levels of miR-20, miR-106a, miR-92a, and miR-203 have been detected in the plasma of SLE patients (68). Moreover, the expression of miR-223 and miR-20 is significantly decreased in SLE patients with active nephritis (68). MiR-20 and miR-92a regulate apoptosis by repressing PTEN and, together with miR-106a, contribute to the control of monocytopoiesis and the regulation of regulatory T cells (30, 69).

MiR-221-5p has been identified as a potential biomarker of renal damage in SLE patients (70). The X-encoded microRNA miR-548m may target PTEN and contribute to SLE pathogenesis (71). Increased levels of miR-224 enhance STAT-1 expression in SLE T cells, promoting lupus nephritis (72). Additionally, Ming Zhao et al. reported that miR-505 is downregulated in T cells of SLE patients (73). All of these studies revealed an important role of X chromosome-encoded miRNAs in various molecular processes involved in disease development.

#### Multiple sclerosis

MS is an immune-mediated neuroinflammatory and demyelinating disease in which the role of miRNAs has been associated with disease onset, activity and duration (74–76). Indeed, miRNAs are highly expressed in cells of the immune system and central nervous system, where they regulate the expression of target genes in different cells. In this regard, the possible effects of miRNAs have been analysed for the MS-associated T cells, B cells, and macrophages (52, 77, 78).

Numerous investigations have detected dysregulated X chromosome-encoded miRNAs in MS patients. For instance, miR-18 and miR-20b are less abundant in relapse remitting MS patients when compared to healthy controls (79). Circulating X-encoded miRNAs such as miR-92a, miR-223 and miR-500, where detected in plasma, serum and cerebrospinal fluid (CSF) and correlate with disease type and severity (80–82). The increased levels of miR-424 in MS patients were associated with a reduction in the levels of CXCL10 and IRF1, as miR-424 is predicted to directly target their respective encoding mRNAs. The decrease in IRF1 and CXCL10 may contribute to a pro-survival phenotype in B cells in MS (83). Furthermore, the abundance of miR-448 also increased significantly in MS, and promoted Th17-mediated immune responses by targeting protein tyrosine phosphatase non-receptor type 2 (PTPN2) (84). In other examples, miR-92a levels negatively correlate with the number of lesions in the posterior inferior lobule and lateral temporal cortex (85), and miR-223 showed decreased serum levels in MS patients compared to healthy controls (86).

These data support the involvement of X-encoded miRNAs in the pathogenesis of ADs, suggesting that sex-linked differences in miRNA expression and regulation may contribute to the heightened susceptibility of women to autoimmunity. In particular, dysregulation of X chromosome-encoded miRNAs appears to influence key immune pathways implicated in RA, SLE, and MS.

### MiRNAs and responses to vaccination

It is widely established that biological sexes differ in their responses to vaccination, although the mechanisms and clinical significance remain uncertain (1, 87). Females exhibit a higher magnitude of immune responses than males from birth to old age for a variety of vaccine candidates, such as inactivated diphtheria, hepatitis B, influenza, rabies, pertussis and pneumococcal vaccines, as well as the live measles vaccine (87). However, more recent evidence shows that sex differences in antibody titres are strongly age-dependent (88, 89), and systems-immunology studies show that these differences extend beyond humoral immunity to include innate and adaptive cell function, cytokine activity, and genetic–epigenetic regulation (90). Females tend to develop more frequent and sometimes more severe vaccine reactions than males, likely reflecting higher inflammatory response (87). They also report more local reactogenicity after influenza and COVID-19 vaccination, while systemic reactions are similar between sexes (87, 91). Conversely, post-authorization data indicate that serious adverse events, including life-threatening conditions and deaths after COVID-19 vaccination, are reported more often in males (91, 92), though these associations do not prove causality.

Sex difference in responses to vaccines could be influenced by multiple factors, including the X chromosome, sex hormones, and other biological mechanisms. In particular, some genes residing on the X chromosome escape XCI and are therefore expressed at higher levels in females, and miRNAs encoded on X chromosome may also contribute to these differences (10, 87). Some X-chromosome miRNAs are expressed more frequently in women due to incomplete XCI, which could contribute to sex differences in immune response to vaccines (21).

Although evidence directly linking X chromosome-residing miRNAs to vaccine responsiveness remains limited, recent studies have begun to uncover sex-dependent patterns of circulating miRNAs expression following immunization. In a cohort of healthcare workers receiving an mRNA-based COVID-19 vaccine, Anticoli et al. reported a sex-dependent miRNA signature, with miR-221-3p, an X-encoded miRNA, targeting molecules of the inflammatory pathways. Interestingly, vaccinated females showed higher miR-221-3p levels than unvaccinated females and vaccinated males, indicating that this upregulation may modulate the innate immune response to vaccine antigens (93). Similarly, the levels of miR-92a-2-5p in serum extracellular vesicles were shown to correlate with both antibody production and post-vaccination inflammatory responses in recipients of vaccines encoding the SARS-CoV-2 spike mRNA (94). Moreover, a recent study demonstrated that X-residing miR-508 and miR-98-5p showed the strongest positive correlation with vaccine-induced IgG antibody response against recombinant Ebola Zaire vaccine (rVSV-ZEBOV), although sex-stratified analyses were not conducted (95). MiR-508 is implicated in different diseases via PI3 K/AKT pathway regulation, affecting cell metabolism, growth, proliferation and survival, while miR-98-5p has been shown to regulate cytokine response via the NF-kB pathway and IL-6 signaling (95).

Collectively, these findings indicate that miRNAs may act as post-transcriptional regulators of vaccine-induced immunity and, interestingly, some of them have been identified as X chromosome resident miRNAs, potentially contributing to the observed sex differences in antibody and T-cell memory responses. However, the current evidence linking miRNAs to vaccine responsiveness remains largely preliminary. Available studies are limited by the smaller proportion of women in vaccine cohorts, the absence of extensive meta-analysis on sex differences, and insufficient statistical power due to limited group size, which together preclude definitive conclusions. In particular, investigations focusing on miRNAs encoded by the X-chromosome and their potential contribution to sex-related differences in long-term vaccine immunity remain scarce. Future research should therefore prioritize longitudinal, well-powered studies integrating sex, age, hormonal status, and functional immune parameters to better define the role of X-linked miRNAs in shaping vaccine-induced immune responses, and to explain individual variability in vaccine efficacy.

## Conclusions

Chromosome X-resident miRNAs represent a pivotal axis in sex-dependent immunity, shaping inflammatory response, susceptibility to ADs, and vaccine responses. The emerging evidence linking X-encoded miRNAs to immune regulation highlights their potential as biomarkers of sex-dependent immune responsiveness and disease susceptibility. Circulating miRNA signatures could serve as minimally invasive indicators of vaccine efficacy and sex-dependent immune responses, shedding light on mechanisms underlying individual differences after vaccination or immune activation.

Beyond their value as biomarkers, miRNAs are being explored as direct therapeutic targets. Several miRNA-based strategies, including mimics and antagomiRs, have entered preclinical and clinical development (96), providing proof-of-concept for their translational potential. Notably, a miR-29 mimic has advanced into clinical trials for fibrotic diseases, based on its ability to limit tissue fibrosis (97, 98), and inhibitors of miR-155 have shown efficacy in preclinical models (99) demonstrating that modulating miRNA activity *in vivo* is feasible and can yield clinically meaningful outcomes. Moreover, they could, in principle, modulate immune reactivity in a sex-tailored manner.

However, it should be noted that research on miRNAs in vaccine responses remains relatively preliminary, with most studies being observational and limited in size or scope. Future research is required to validate the predictive and mechanistic roles of X chromosome-encoded miRNAs in vaccine-induced immunity. Such investigations could clarify the potential of miRNAs as modulators of sex-dependent vaccine efficacy and immune reactivity, while addressing current limitations in evidence.

Finally, translating these findings into clinical practice will require longitudinal, sex- and age-stratified studies that integrate genomic, hormonal, and epigenetic variables to validate the predictive and mechanistic value of X chromosome-encoded miRNAs. Critically, evaluations must consider the influence of age and distinct life stages in women, from infancy to menopause and older age, to avoid methodological biases and accurately capture sex-dependent immune dynamics (100, 101). Understanding the regulation of X chromosome-encoded miRNAs in this context promises to advance precision medicine approaches for vaccination, inflammatory disorders, and autoimmune diseases.

## References

[1] Klein SL Flanagan KL. Sex differences in immune responses. Nat Rev Immunol. (2016) 16(10):626–38. 10.1038/nri.2016.90 27546235

[2] Migeon BR. Why females are mosaics, x-chromosome inactivation, and sex differences in disease. Gend Med. (2007) 4(2):97–105. 10.1016/S1550-8579(07)80024-6 17707844

[3] Rio P Caldarelli M Miccoli E Guazzarotti G Gasbarrini A Gambassi G Sex differences in immune responses to infectious diseases: the role of genetics, hormones, and aging. Diseases. (2025) 13(6):1–24. 10.3390/diseases13060179 PMC12191887 40558589

[4] Miquel CH Faz-Lopez B Guéry JC. Influence of X chromosome in sex-biased autoimmune diseases. J Autoimmun. (2023) 137:102992. 10.1016/j.jaut.2023.102992 36641351

[5] Margarita V Lodde V Rappelli P Doro L Montella A Fiori PL The different innate immune response to infections in males and females emerges before birth. Life Sci. (2025) 369:123521. 10.1016/j.lfs.2025.123521 40044031

[6] Migeon BR. X-linked diseases: susceptible females. Genet Med. (2020) 22(7):1156–74. 10.1038/s41436-020-0779-4 32284538 PMC7332419

[7] Bianchi I Lleo A Gershwin ME Invernizzi P. The X chromosome and immune associated genes. J Autoimmun. (2012) 38(2–3):J187–92. 10.1016/j.jaut.2011.11.012 22178198

[8] Mousavi MJ Mahmoudi M Ghotloo S. Escape from X chromosome inactivation and female bias of autoimmune diseases. Mol Med. (2020) 26(1):1–20. 10.1186/s10020-020-00256-1 PMC7727198 33297945

[9] Brockdorff N Turner BM. Dosage compensation in mammals. Cold Spring Harb Perspect Biol. (2015) 7(3):a019406. 10.1101/cshperspect.a019406 25731764 PMC4355265

[10] Ercan S. Mechanisms of X chromosome dosage compensation. J Genomics. (2014) 3:1–19. 10.7150/jgen.10404 PMC4303597 25628761

[11] Pinheiro I Dejager L Libert C. X-chromosome-located microRNAs in immunity: might they explain male/female differences?: the X chromosome-genomic context may affect X-located miRNAs and downstream signaling, thereby contributing to the enhanced immune response of females. BioEssays. (2011) 33(11):791–802. 10.1002/bies.201100047 21953569

[12] Lee Y Ahn C Han J Choi H Kim J Yim J The nuclear RNase III drosha initiates microRNA processing. Nature. (2003) 425(6956):415–9. 10.1038/nature01957 14508493

[13] Gregory RI Yan KP Amuthan G Chendrimada T Doratotaj B Cooch N The microprocessor complex mediates the genesis of microRNAs. Nature. (2004) 432(7014):235–40. 10.1038/nature03120 15531877

[14] Okada C Yamashita E Lee SJ Shibata S Katahira J Nakagawa A A high-resolution structure of the pre-microrna nuclear export machinery. Science. (2009) 326(5957):1275–9. 10.1126/science.1178705 19965479

[15] Tang G. siRNA and miRNA: an insight into RISCs. Trends Biochem Sci. (2005) 30(2):106–14. 10.1016/j.tibs.2004.12.007 15691656

[16] Fabian MR Sonenberg N. The mechanics of miRNA-mediated gene silencing: a look under the hood of miRISC. Nat Struct Mol Biol. (2012) 19(6):586–93. 10.1038/nsmb.2296 22664986

[17] Naeli P Winter T Hackett AP Alboushi L Jafarnejad SM. The intricate balance between microRNA-induced mRNA decay and translational repression. FEBS J. (2023) 290(10):2508–24. 10.1111/febs.16422 35247033

[18] Bhaskaran M Mohan M. MicroRNAs: history, biogenesis, and their evolving role in animal development and disease. Vet Pathol. (2014) 51(4):759–74. 10.1177/0300985813502820 24045890 PMC4013251

[19] Di Palo A Siniscalchi C Salerno M Russo A Gravholt CH Potenza N. What microRNAs could tell US about the human X chromosome. Cell Mol Life Sci. (2020) 77(20):4069–80. 10.1007/s00018-020-03526-7 32356180 PMC7854456

[20] Ambros V Bartel B Bartel DP Burge CB Carrington JC Chen X A uniform system for microRNA annotation. RNA. (2003) 9(3):277–9. 10.1261/rna.2183803 12592000 PMC1370393

[21] Song R Ro S Michaels JD Park C Mccarrey JR Yan W. Many X-linked microRNAs escape meiotic sex chromosome inactivation. Nat Genet. (2009) 41(4):488–93. 10.1038/ng.338 19305411 PMC2723799

[22] Matarrese P Tieri P Anticoli S Ascione B Conte M Franceschi C Correction: x-chromosome-linked miR548am-5p is a key regulator of sex disparity in the susceptibility to mitochondria-mediated apoptosis. Cell Death Dis. (2019) 10(9):673. 10.1038/s41419-019-1888-3 31511496 PMC6739406

[23] Chureau C Chantalat S Romito A Galvani A Duret L Avner P Ftx is a non-coding RNA which affects Xist expression and chromatin structure within the X-inactivation center region. Hum Mol Genet. (2011) 20(4):705–18. 10.1093/hmg/ddq516 21118898

[24] Lou S Tihagam D Wasko UN Equbal Z Venkatesan S Braczyk K Targeting microRNA-dependent control of X chromosome inactivation improves the rett syndrome phenotype. Nat Commun. (2025) 16(1):6169. 10.1038/s41467-025-61092-7 40615387 PMC12227778

[25] Vlachos IS Zagganas K Paraskevopoulou MD Georgakilas G Karagkouni D Vergoulis T DIANA-miRPath v3.0: deciphering microRNA function with experimental support. Nucleic Acids Res. (2015) 43(W1):W460–6. 10.1093/nar/gkv403 25977294 PMC4489228

[26] Baltimore D Boldin MP O’Connell RM Rao DS Taganov KD. MicroRNAs: new regulators of immune cell development and function. Nat Immunol. (2008) 9(8):839–45. 10.1038/ni.f.209 18645592

[27] Masaki S Ohtsuka R Abe Y Muta K Umemura T. Expression patterns of microRNAs 155 and 451 during normal human erythropoiesis. Biochem Biophys Res Commun. (2007) 364(3):509–14. 10.1016/j.bbrc.2007.10.077 17964546

[28] Johnnidis JB Harris MH Wheeler RT Stehling-Sun S Lam MH Kirak O Regulation of progenitor cell proliferation and granulocyte function by microRNA-223. Nature. (2008) 451(7182):1125–9. 10.1038/nature06607 18278031

[29] Zhang N Fu L Bu Y Yao Y Wang Y. Downregulated expression of miR-223 promotes toll-like receptor-activated inflammatory responses in macrophages by targeting RhoB. Mol Immunol. (2017) 91:42–8. 10.1016/j.molimm.2017.08.026 28881218

[30] Fontana L Pelosi E Greco P Racanicchi S Testa U Liuzzi F MicroRNAs 17-5p-20a-106a control monocytopoiesis through AML1 targeting and M-CSF receptor upregulation. Nat Cell Biol. (2007) 9(7):775–87. 10.1038/ncb1613 17589498

[31] Kästle M Bartel S Geillinger-Kästle K Irmler M Beckers J Ryffel B microRNA cluster 106a∼363 is involved in T helper 17 cell differentiation. Immunology. (2017) 152(3):402–13. 10.1111/imm.12775 28617945 PMC5629441

[32] Landais S Landry S Legault P Rassart E. Oncogenic potential of the miR-106-363 cluster and its implication in human T-cell leukemia. Cancer Res. (2007) 67(12):5699–707. 10.1158/0008-5472.CAN-06-4478 17575136

[33] Forrest ARR Kanamori-Katayama M Tomaru Y Lassmann T Ninomiya N Takahashi Y Induction of microRNAs, mir-155, mir-222, mir-424 and mir-503, promotes monocytic differentiation through combinatorial regulation. Leukemia. (2010) 24(2):460–6. 10.1038/leu.2009.246 19956200

[34] Felli N Fontana L Pelosi E Botta R Bonci D Facchiano F MicroRNAs 221 and 222 inhibit normal erythropoiesis and erythroleukemic cell growth via kit receptor down-modulation. Proc Natl Acad Sci U S A. (2005) 102(50):18081–6. 10.1073/pnas.0506216102 16330772 PMC1312381

[35] Grigoryev YA Kurian SM Hart T Nakorchevsky AA Campbell D Head SR MiRNA post-transcriptional modification dynamics in T cell activation. iScience. (2021) 24(6):102530. 10.1016/j.isci.2021.102530 34142042 PMC8188497

[36] Sharma A Kumar M Aich J Hariharan M Brahmachari SK Agrawal A Posttranscriptional regulation of interleukin-10 expression by hsa-miR-106a. Proc Natl Acad Sci U S A. (2009) 106(14):5761–6. 10.1073/pnas.0808743106 19307576 PMC2659714

[37] Zhou H Sun C Li C Hua S Li F Li R The MicroRNA-106a/20b strongly enhances the antitumour immune responses of dendritic cells pulsed with glioma stem cells by targeting STAT3. J Immunol Res. (2022) 15:9721028. 10.1155/2022/9721028 PMC9499788 36157880

[38] Gantier MP Stunden HJ McCoy CE Behlke MA Wang D Kaparakis-Liaskos M A miR-19 regulon that controls NF-*κ*B signaling. Nucleic Acids Res. (2012) 40(16):8048–58. 10.1093/nar/gks521 22684508 PMC3439911

[39] Liu Y Chen Q Song Y Lai L Wang J Yu H MicroRNA-98 negatively regulates IL-10 production and endotoxin tolerance in macrophages after LPS stimulation. FEBS Lett. (2011) 585(12):1963–8. 10.1016/j.febslet.2011.05.029 21609717

[40] Wang L Wang FS Gershwin ME. Human autoimmune diseases: a comprehensive update. J Intern Med. (2015) 278(4):369–95. 10.1111/joim.12395 26212387

[41] Angum F Khan T Kaler J Siddiqui L Hussain A. The prevalence of autoimmune disorders in women: a narrative review. Cureus. (2020) 12(5):e8094. 10.7759/cureus.8094 32542149 PMC7292717

[42] Ortona E Pierdominici M Maselli A Veroni CF Shoenfeld Y. Sex-based differences in autoimmune diseases. Ann Ist Super Sanità. (2016) 52(2):205–2012. 10.4415/ANN_16_02_12 27364395

[43] Bueno MJ Malumbres M. MicroRNAs and the cell cycle. Biochim Biophys Acta - Mol Basis Dis. (2011) 1812(5):592–601. 10.1016/j.bbadis.2011.02.002 21315819

[44] Su Z Yang Z Xu Y Chen Y Yu Q. MicroRNAs in apoptosis, autophagy and necroptosis. Oncotarget. (2015) 6(11):8474–90. 10.18632/oncotarget.3523 25893379 PMC4496162

[45] Munk R Panda AC Grammatikakis I Gorospe M Abdelmohsen K. Senescence-associated MicroRNAs. Int Rev Cell Mol Biol. (2018) 334:177–205. 10.1016/bs.ircmb.2017.03.008 PMC8436595 28838538

[46] Long H Wang X Chen Y Wang L Zhao M Lu Q. Dysregulation of microRNAs in autoimmune diseases: pathogenesis, biomarkers and potential therapeutic targets. Cancer Lett. (2018) 428:90–103. 10.1016/j.canlet.2018.04.016 29680223

[47] Zhang L Wu H Zhao M Chang C Lu Q. Clinical significance of miRNAs in autoimmunity. J Autoimmun. (2020) 109(139):102438. 10.1016/j.jaut.2020.102438 32184036

[48] Gaál Z. Role of microRNAs in immune regulation with translational and clinical applications. Int J Mol Sci. (2024) 25(3):1942. 10.3390/ijms25031942 38339220 PMC10856342

[49] Salvi V Gianello V Tiberio L Sozzani S Bosisio D. Cytokine targeting by miRNAs in autoimmune diseases. Front Immunol. (2019) 10:1–10. 10.3389/fimmu.2019.00015 30761124 PMC6361839

[50] Schell SL Rahman ZSM. miRNA-mediated control of B cell responses in immunity and SLE. Front Immunol. (2021) 12:1–20. 10.3389/fimmu.2021.683710 PMC8165268 34079558

[51] Banerjee S Thompson WE Chowdhury I. Emerging roles of microRNAs in the regulation of toll-like receptor (TLR)-signaling. Front Biosci - Landmark. (2021) 26(4):771–96. 10.2741/4917 PMC9550351 33049693

[52] Holvoet P. miRNAs and T cell-mediated immune response in disease. Yale J Biol Med. (2025) 98(2):187–202. 10.59249/PAYJ6872 40589938 PMC12204035

[53] Ebrahimiyan H Ahmadzadeh A. miRNAs and rheumatoid arthritis: new update in expression pattern and pathogenicity. Rheumatol Res. (2021) 6(3):123–37. 10.1002/iid3.914

[54] Peng X Wang Q Li W Ge G Peng J Xu Y Comprehensive overview of microRNA function in rheumatoid arthritis. Bone Res. (2023) 11(1):8. 10.1038/s41413-023-00244-1 36690624 PMC9870909

[55] Filková M Aradi B Šenolt L Ospelt C Vettori S Mann H Association of circulating miR-223 and miR-16 with disease activity in patients with early rheumatoid arthritis. Ann Rheum Dis. (2014) 73(10):1898–904. 10.1136/annrheumdis-2012-202815 23897768 PMC4173742

[56] Shibuya H Nakasa T Adachi N Nagata Y Ishikawa M Deie M Overexpression of microRNA-223 in rheumatoid arthritis synovium controls osteoclast differentiation. Mod Rheumatol. (2013) 23(4):674–85. 10.3109/s10165-012-0710-1 22903258

[57] Khalifa O Pers YM Ferreira R Sénéchal A Jorgensen C Apparailly F X-linked miRNAs associated with gender differences in rheumatoid arthritis. Int J Mol Sci. (2016) 17(11):1852. 10.3390/ijms17111852 27834806 PMC5133852

[58] Philippe L Alsaleh G Suffert G Meyer A Georgel P Sibilia J TLR2 expression is regulated by MicroRNA miR-19 in rheumatoid fibroblast-like synoviocytes. J Immunol. (2012) 188(1):454–61. 10.4049/jimmunol.1102348 22105995

[59] Philippe L Alsaleh G Bahram S Pfeffer S Georgel P. The miR-17∼92 cluster: a key player in the control of inflammation during rheumatoid arthritis. Front Immunol. (2013) 4:1–5. 10.3389/fimmu.2013.00070 23516027 PMC3601326

[60] Li Z Zhao W Wang M Hussain MZ Mahjabeen I. Role of microRNAs deregulation in initiation of rheumatoid arthritis: a retrospective observational study. Med (United States). (2024) 103(3):E36595. 10.1097/MD.0000000000036595 PMC10798721 38241560

[61] Yu FY Xie CQ Jiang CL Sun JT Feng HC Li C MiR-92a inhibits fibroblast-like synoviocyte proliferation and migration in rheumatoid arthritis by targeting AKT2. J Biosci. (2018) 43(5):911–9. 10.1007/s12038-018-9803-0 30541951

[62] Ruedel A Dietrich P Schubert T Hofmeister S Hellerbrand C Bosserhoff AK. Expression and function of microRNA-188-5p in activated rheumatoid arthritis synovial fibroblasts. Int J Clin Exp Pathol. (2015) 8(6):6607–16. 26261542 PMC4525876

[63] Abo ElAtta AS Ali YBM Bassyouni IH Talaat RM. Upregulation of miR-221/222 expression in rheumatoid arthritis (RA) patients: correlation with disease activity. Clin Exp Med. (2019) 19(1):47–53. 10.1007/s10238-018-0524-3 30132091

[64] Yang S Yang Y. Downregulation of microRNA-221 decreases migration and invasion in fibroblast-like synoviocytes in rheumatoid arthritis. Mol Med Rep. (2015) 12(2):2395–401. 10.3892/mmr.2015.3642 25891943

[65] Dai Y Huang YS Tang M Lv TY Hu CX Tan YH Microarray analysis of microRNA expression in peripheral blood cells of systemic lupus erythematosus patients. Lupus. (2007) 16(12):939–46. 10.1177/0961203307084158 18042587

[66] Sui W Liu F Chen J Ou M Dai Y. Microarray technology for analysis of microRNA expression in renal biopsies of lupus nephritis patients. Methods Mol Biol. (2014) 1134:211–20. 10.1007/978-1-4939-0326-9_16 24497365

[67] Chen JQ Papp G Póliska S Szabó K Tarr T Bálint BL MicroRNA expression profiles identify disease-specific alterations in systemic lupus erythematosus and primary sjögren’s syndrome. PLoS One. (2017) 12(3):e0174585. 10.1371/journal.pone.0174585 28339495 PMC5365120

[68] Carlsen AL Schetter AJ Nielsen CT Lood C Knudsen S Voss A Circulating microRNA expression profiles associated with systemic lupus erythematosus. Arthritis Rheum. (2013) 65(5):1324–34. 10.1002/art.37890 23401079 PMC6662589

[69] Jiang S Li C Olive V Lykken E Feng F Sevilla J Molecular dissection of the miR-17-92 cluster’s critical dual roles in promoting Th1 responses and preventing inducible treg differentiation. Blood. (2011) 118(20):5487–97. 10.1182/blood-2011-05-355644 21972292 PMC3217351

[70] Navarro-Quiroz E Pacheco-Lugo L Lorenzi H Díaz-Olmos Y Almendrales L Rico E High-throughput sequencing reveals circulating miRNAs as potential biomarkers of kidney damage in patients with systemic lupus erythematosus. PLoS One. (2016) 11(11):e0166202. 10.1371/journal.pone.0166202 27835701 PMC5106044

[71] Yang X Shi L Zheng X Liu XQJ. Modulation of miR-548m encoded by X chromosome on the PTEN pathway in systemic lupus erythematosus. Clin Exp Rheumatol. (2022) 40(1):56–63. 10.55563/clinexprheumatol/yjsbqm 33635226

[72] Lu MC Lai NS Chen HC Yu HC Huang KY Tung CH Decreased microRNA(miR)-145 and increased miR-224 expression in T cells from patients with systemic lupus erythematosus involved in lupus immunopathogenesis. Clin Exp Immunol. (2013) 171(1):91–9. 10.1111/j.1365-2249.2012.04676.x 23199328 PMC3530100

[73] Zhao M Liu S Luo S Wu H Tang M Cheng W DNA methylation and mRNA and microRNA expression of SLE CD4+ T cells correlate with disease phenotype. J Autoimmun. (2014) 54:127–36. 10.1016/j.jaut.2014.07.002 25091625

[74] Tufekci KU Oner MG Genc S Genc K. MicroRNAs and multiple sclerosis. Autoimmune Dis. (2010) 2011:807426. 10.4061/2011/807426 21188194 PMC3003960

[75] Huang Q Xiao B Ma X Qu M Li Y Nagarkatti P MicroRNAs associated with the pathogenesis of multiple sclerosis. J Neuroimmunol. (2016) 295:148–61. 10.1016/j.jneuroim.2016.04.014 27235360

[76] Al-Temaimi R Alshammari N Alroughani R. Analysis of potential microRNA biomarkers for multiple sclerosis. Exp Mol Pathol. (2024) 137:104903. 10.1016/j.yexmp.2024.104903 38772208

[77] Liu G Abraham E. MicroRNAs in immune response and macrophage polarization. Arterioscler Thromb Vasc Biol. (2013) 33(2):170–7. 10.1161/ATVBAHA.112.300068 23325473 PMC3549532

[78] Roy U Raghavan SC. Regulation of B-cell development and differentiation by microRNAs during immune response and their implications in immunological disorders. J Immunol. (2025) 214(12):vkaf203. 10.1093/jimmun/vkaf203 40803332

[79] Niwald M Migdalska-Sęk M Brzeziańska-Lasota E Miller E. Evaluation of selected MicroRNAs expression in remission phase of multiple sclerosis and their potential link to cognition, depression, and disability. J Mol Neurosci. (2017) 63(3-4):275–82. 10.1007/s12031-017-0977-y 29043654

[80] Gandhi R Healy B Gholipour T Egorova S Musallam A Hussain MS Circulating microRNAs as biomarkers for disease staging in multiple sclerosis. Ann Neurol. (2013) 73(6):729–40. 10.1002/ana.23880 23494648

[81] Fenoglio C Ridolfi E Cantoni C De Riz M Bonsi R Serpente M Decreased circulating miRNA levels in patients with primary progressive multiple sclerosis. Mult Scler J. (2013) 19(14):1938–42. 10.1177/1352458513485654 24277735

[82] Kacperska MJ Jastrzebski K Tomasik B Walenczak J Konarska-Krol M Glabinski A. Selected extracellular microRNA as potential biomarkers of multiple sclerosis activity—preliminary study. J Mol Neurosci. (2015) 56(1):154–63. 10.1007/s12031-014-0476-3 25487315 PMC4382531

[83] Annibali V Umeton R Palermo A Severa M Etna MP Giglio S Analysis of coding and non-coding transcriptome of peripheral B cells reveals an altered interferon response factor (IRF)-1 pathway in multiple sclerosis patients. J Neuroimmunol. (2018) 324:165–71. 10.1016/j.jneuroim.2018.09.005 30270021

[84] Wu R He Q Chen H Xu M Zhao N Xiao Y MicroRNA-448 promotes multiple sclerosis development through induction of Th17 response through targeting protein tyrosine phosphatase non-receptor type 2 (PTPN2). Biochem Biophys Res Commun. (2017) 486(3):759–66. 10.1016/j.bbrc.2017.03.115 28342869

[85] Geiger L Orsi G Cseh T Gombos K Illés Z Czéh B. Circulating microRNAs correlate with structural and functional MRI parameters in patients with multiple sclerosis. Front Mol Neurosci. (2023) 16(10):1173212. 10.3389/fnmol.2023.1173212 37881368 PMC10597671

[86] Zabalza A Pappolla A Comabella M Montalban X Malhotra S. MiRNA-based therapeutic potential in multiple sclerosis. Front Immunol. (2024) 15(29):1441733. 10.3389/fimmu.2024.1441733 39267760 PMC11390414

[87] Flanagan KL Fink AL Plebanski M Klein SL. Sex and gender differences in the outcomes of vaccination over the life course. Annu Rev Cell Dev Biol. (2017) 33:577–99. 10.1146/annurev-cellbio-100616-060718 28992436

[88] Kuo H Shapiro JR Dhakal S Morgan R Fink AL Liu H Sex-specific effects of age and body mass index on antibody responses to seasonal influenza vaccines in healthcare workers. Vaccine. (2022) 40(11):1634–42. 10.1016/j.vaccine.2021.02.047 33678455 PMC8417149

[89] Hoshida S Koeda N Hattori H Tanaka M Tanaka I Fukui H Age - and sex - based changes in spike protein antibody status after SARS - CoV - 2 vaccination and effect of past - infection in healthcare workers in Osaka. BMC Infect Dis. (2022) 22(1):709. 10.1186/s12879-022-07695-7 36028796 PMC9412794

[90] Furman D Hejblum BP Simon N Jojic V Dekker CL Thiébaut R Systems analysis of sex differences reveals an immunosuppressive role for testosterone in the response to influenza vaccination. Proc Natl Acad Sci U S A. (2014) 111(2):869–74. 10.1073/pnas.1321060111 24367114 PMC3896147

[91] Yin A Wang N Shea PJ Rosser EN Kuo H Shapiro JR Sex and gender differences in adverse events following influenza and COVID-19 vaccination. Biol Sex Differ. (2024) 15(1):50. 10.1186/s13293-024-00625-z 38890702 PMC11184791

[92] Janekrongtham C Salazar M Doung-ngern P. Sex differences in serious adverse events reported following booster doses of COVID-19 vaccination in Thailand : a countrywide nested unmatched case-control study. Vaccines (Basel). (2023) 11(12):1772. 10.3390/vaccines11121772 38140176 PMC10747632

[93] Anticoli S Dorrucci M Iessi E Zaffina S Carsetti R Vonesch N Profile of selected microRNAs as markers of sex-specific anti-S/RBD response to COVID-19 mRNA vaccine in health care workers. Int J Mol Sci. (2025) 26(15):7636. 10.3390/ijms26157636 40806764 PMC12346932

[94] Miyashita Y Yoshida T Takagi Y Tsukamoto H Takashima K Kouwaki T Circulating extracellular vesicle microRNAs associated with adverse reactions, proin fl ammatory cytokine, and antibody production after COVID-19 vaccination. npj Vaccines. (2022) 7(1):16. 10.1038/s41541-022-00439-3 35136071 PMC8826357

[95] Vianello E Persson J Andersson B van Veen S Dias TL Santoro F Global blood miRNA profiling unravels early signatures of immunogenicity of ebola vaccine rVSV*Δ*G-ZEBOV-GP. iScience. (2023) 26(12):108574. 10.1016/j.isci.2023.108574 38162033 PMC10755791

[96] Romano G Acunzo M Nana-Sinkam P. Micrornas as novel therapeutics in cancer. Cancers (Basel). (2021) 13(7):1526. 10.3390/cancers13071526 33810332 PMC8037786

[97] Chioccioli M Roy S Newell R Pestano L Dickinson B Rigby K A lung targeted miR-29 mimic as a therapy for pulmonary fibrosis. eBioMedicine. (2022) 85:104304. 10.1016/j.ebiom.2022.104304 36265417 PMC9587275

[98] McDaniel G Li Y Driscoll TP. miR29a-loaded extracellular vesicles derived from human mesenchymal stem cells inhibit fibrotic and inflammatory signaling. ACS Omega. (2025) 10(42):49536–44. 10.1021/acsomega.5c03490 41179181 PMC12572971

[99] Singh A Dashynam M Chim B Escobar TM Liu X Hu X Identification of small molecule inhibitors of a Mir155 transcriptional reporter in Th17 cells. Sci Rep. (2021) 11(1):1–14. 10.1038/s41598-021-90944-7 34075120 PMC8169650

[100] Franconi F Rosano G Campesi I. Need for gender-specific pre-analytical testing : the dark side of the moon in laboratory testing. Int J Cardiol. (2015) 179:514–35. 10.1016/j.ijcard.2014.11.019 25465806

[101] Franconi F Campesi I. Sex impact on biomarkers, pharmacokinetics and pharmacodynamics. Curr Med Chem. (2017) 24(24):2561–75. 10.2174/0929867323666161003124616 27697075

