# Supplementary material for: X chromosome-encoded microRNAs in immune regulation: sex differences and clinical implications
Source: Front Glob Womens Health. 2026 Feb 19;7:1758961. doi: 10.3389/fgwh.2026.1758961 (PMC12960141; doi:10.3389/fgwh.2026.1758961)
Supplement: Supplementary file 1 [file Table1.docx]

**Supplemental table 1**: List of miRNAs sequence mapping on X chromosome based on miRBase database

| **Name** | **Accession** | **Chr** | **Start** | **End** | **Strand** | **Confidence** |
| --- | --- | --- | --- | --- | --- | --- |
| hsa-let-7f-2 | MI0000068 | chrX | 53557192 | 53557274 | - | High |
| hsa-mir-19b-2 | MI0000075 | chrX | 134169671 | 134169766 | - | High |
| hsa-mir-92a-2 | MI0000094 | chrX | 134169538 | 134169612 | - | High |
| hsa-mir-98 | MI0000100 | chrX | 53556223 | 53556341 | - | High |
| hsa-mir-105-1 | MI0000111 | chrX | 152392219 | 152392299 | - | High |
| hsa-mir-105-2 | MI0000112 | chrX | 152394412 | 152394492 | - | High |
| hsa-mir-106a | MI0000113 | chrX | 134170198 | 134170278 | - | High |
| hsa-mir-221 | MI0000298 | chrX | 45746157 | 45746266 | - | High |
| hsa-mir-222 | MI0000299 | chrX | 45747015 | 45747124 | - | High |
| hsa-mir-223 | MI0000300 | chrX | 66018870 | 66018979 | + | High |
| hsa-mir-224 | MI0000301 | chrX | 151958578 | 151958658 | - | High |
| hsa-mir-188 | MI0000484 | chrX | 50003503 | 50003588 | + | High |
| hsa-mir-361 | MI0000760 | chrX | 85903636 | 85903707 | - | High |
| hsa-mir-362 | MI0000762 | chrX | 50008964 | 50009028 | + | High |
| hsa-mir-363 | MI0000764 | chrX | 134169378 | 134169452 | - | High |
| hsa-mir-374a | MI0000782 | chrX | 74287286 | 74287357 | - | High |
| hsa-mir-424 | MI0001446 | chrX | 134546614 | 134546711 | - | High |
| hsa-mir-18b | MI0001518 | chrX | 134170041 | 134170111 | - | High |
| hsa-mir-20b | MI0001519 | chrX | 134169809 | 134169877 | - | High |
| hsa-mir-450a-1 | MI0001652 | chrX | 134540341 | 134540431 | - | High |
| hsa-mir-452 | MI0001733 | chrX | 151959628 | 151959712 | - | High |
| hsa-mir-500a | MI0003184 | chrX | 50008431 | 50008514 | + | High |
| hsa-mir-501 | MI0003185 | chrX | 50009722 | 50009805 | + | High |
| hsa-mir-502 | MI0003186 | chrX | 50014598 | 50014683 | + | High |
| hsa-mir-450a-2 | MI0003187 | chrX | 134540508 | 134540607 | - | High |
| hsa-mir-503 | MI0003188 | chrX | 134546328 | 134546398 | - | High |
| hsa-mir-504 | MI0003189 | chrX | 138667711 | 138667793 | - | High |
| hsa-mir-513a-1 | MI0003191 | chrX | 147213463 | 147213591 | - | High |
| hsa-mir-513a-2 | MI0003192 | chrX | 147225826 | 147225952 | - | High |
| hsa-mir-506 | MI0003193 | chrX | 147230720 | 147230843 | - | High |
| hsa-mir-508 | MI0003195 | chrX | 147236913 | 147237027 | - | High |
| hsa-mir-509-1 | MI0003196 | chrX | 147260532 | 147260625 | - | High |
| hsa-mir-510 | MI0003197 | chrX | 147272335 | 147272408 | - | High |
| hsa-mir-514a-1 | MI0003198 | chrX | 147279247 | 147279344 | - | High |
| hsa-mir-514a-2 | MI0003199 | chrX | 147281943 | 147282030 | - | High |
| hsa-mir-514a-3 | MI0003200 | chrX | 147284641 | 147284728 | - | High |
| hsa-mir-532 | MI0003205 | chrX | 50003148 | 50003238 | + | High |
| hsa-mir-545 | MI0003516 | chrX | 74287104 | 74287209 | - | High |
| hsa-mir-651 | MI0003666 | chrX | 8126965 | 8127061 | + | High |
| hsa-mir-652 | MI0003667 | chrX | 110055329 | 110055426 | + | High |
| hsa-mir-660 | MI0003684 | chrX | 50013241 | 50013337 | + | High |
| hsa-mir-421 | MI0003685 | chrX | 74218377 | 74218461 | - | High |
| hsa-mir-542 | MI0003686 | chrX | 134541341 | 134541437 | - | High |
| hsa-mir-767 | MI0003763 | chrX | 152393421 | 152393529 | - | High |
| hsa-mir-766 | MI0003836 | chrX | 119646738 | 119646848 | - | High |
| hsa-mir-1298 | MI0003938 | chrX | 114715233 | 114715344 | + | High |
| hsa-mir-509-2 | MI0005530 | chrX | 147258760 | 147258850 | - | High |
| hsa-mir-450b | MI0005531 | chrX | 134540185 | 134540262 | - | High |
| hsa-mir-888 | MI0005537 | chrX | 145994784 | 145994860 | - | High |
| hsa-mir-374b | MI0005566 | chrX | 74218547 | 74218618 | - | High |
| hsa-mir-509-3 | MI0005717 | chrX | 147259652 | 147259726 | - | High |
| hsa-mir-1277 | MI0006419 | chrX | 118386394 | 118386471 | + | High |
| hsa-mir-513b | MI0006648 | chrX | 147199044 | 147199127 | - | High |
| hsa-mir-513c | MI0006649 | chrX | 147189704 | 147189787 | - | High |
| hsa-mir-1912 | MI0008333 | chrX | 114651544 | 114651623 | + | High |
| hsa-mir-2114 | MI0010633 | chrX | 150228004 | 150228083 | + | High |
| hsa-mir-514b | MI0014251 | chrX | 147250151 | 147250230 | - | High |
| hsa-mir-676 | MI0016436 | chrX | 70022857 | 70022923 | + | High |
| hsa-mir-548am | MI0016904 | chrX | 16627012 | 16627085 | - | High |
| hsa-mir-664b | MI0019134 | chrX | 154768596 | 154768656 | + | High |
| hsa-mir-548ax | MI0019286 | chrX | 11318614 | 11318686 | - | High |
| hsa-mir-892c | MI0022560 | chrX | 145992750 | 145992826 | - | High |
| hsa-mir-4536-2 | MI0019149 | chrX | 55451495 | 55451582 | + | Low |
| hsa-mir-325 | MI0000824 | chrX | 77005404 | 77005501 | - | - |
| hsa-mir-384 | MI0001145 | chrX | 76919273 | 76919360 | - | - |
| hsa-mir-448 | MI0001637 | chrX | 114823454 | 114823564 | + | - |
| hsa-mir-505 | MI0003190 | chrX | 139924148 | 139924231 | - | - |
| hsa-mir-507 | MI0003194 | chrX | 147230984 | 147231077 | - | - |
| hsa-mir-1264 | MI0003758 | chrX | 114652655 | 114652723 | + | - |
| hsa-mir-1468 | MI0003782 | chrX | 63786002 | 63786087 | - | - |
| hsa-mir-764 | MI0003944 | chrX | 114639435 | 114639519 | + | - |
| hsa-mir-891a | MI0005524 | chrX | 146027794 | 146027872 | - | - |
| hsa-mir-892a | MI0005528 | chrX | 145996669 | 145996743 | - | - |
| hsa-mir-890 | MI0005533 | chrX | 145994275 | 145994351 | - | - |
| hsa-mir-891b | MI0005534 | chrX | 146001053 | 146001131 | - | - |
| hsa-mir-892b | MI0005538 | chrX | 145997198 | 145997274 | - | - |
| hsa-mir-934 | MI0005756 | chrX | 136550878 | 136550960 | + | - |
| hsa-mir-1184-1 | MI0006277 | chrX | 154887360 | 154887458 | - | - |
| hsa-mir-548f-5 | MI0006378 | chrX | 32641474 | 32641559 | - | - |
| hsa-mir-548m | MI0006400 | chrX | 95063141 | 95063226 | - | - |
| hsa-mir-548i-4 | MI0006424 | chrX | 84225752 | 84225828 | - | - |
| hsa-mir-1321 | MI0006652 | chrX | 85835780 | 85835858 | + | - |
| hsa-mir-320d-2 | MI0008192 | chrX | 140926160 | 140926231 | - | - |
| hsa-mir-1911 | MI0008332 | chrX | 114763184 | 114763263 | + | - |
| hsa-mir-718 | MI0012489 | chrX | 154019920 | 154019989 | - | - |
| hsa-mir-3202-1 | MI0014252 | chrX | 153981097 | 153981177 | + | - |
| hsa-mir-3202-2 | MI0014253 | chrX | 153981098 | 153981176 | - | - |
| hsa-mir-4329 | MI0015901 | chrX | 112780718 | 112780788 | - | - |
| hsa-mir-4330 | MI0015902 | chrX | 151168222 | 151168326 | + | - |
| hsa-mir-500b | MI0015903 | chrX | 50010672 | 50010750 | + | - |
| hsa-mir-4328 | MI0015904 | chrX | 78901194 | 78901249 | - | - |
| hsa-mir-1184-2 | MI0015971 | chrX | 155383100 | 155383198 | - | - |
| hsa-mir-1184-3 | MI0015972 | chrX | 155457517 | 155457615 | + | - |
| hsa-mir-23c | MI0016010 | chrX | 20017088 | 20017187 | - | - |
| hsa-mir-3672 | MI0016073 | chrX | 121370972 | 121371053 | + | - |
| hsa-mir-3690-1 | MI0016091 | chrX | 1293918 | 1293992 | + | - |
| hsa-mir-3915 | MI0016420 | chrX | 32583656 | 32583752 | - | - |
| hsa-mir-3937 | MI0016593 | chrX | 39661216 | 39661321 | + | - |
| hsa-mir-374c | MI0016684 | chrX | 74218549 | 74218618 | + | - |
| hsa-mir-548aj-2 | MI0016815 | chrX | 38023895 | 38023986 | - | - |
| hsa-mir-1587 | MI0016905 | chrX | 39837561 | 39837613 | + | - |
| hsa-mir-4536-1 | MI0016906 | chrX | 55451495 | 55451582 | - | - |
| hsa-mir-548an | MI0016907 | chrX | 106639814 | 106639896 | + | - |
| hsa-mir-3978 | MI0016996 | chrX | 110082118 | 110082218 | + | - |
| hsa-mir-4767 | MI0017408 | chrX | 7147860 | 7147937 | + | - |
| hsa-mir-4768 | MI0017409 | chrX | 17425881 | 17425954 | + | - |
| hsa-mir-4769 | MI0017410 | chrX | 47587429 | 47587505 | + | - |
| hsa-mir-4770 | MI0017411 | chrX | 6383906 | 6383963 | - | - |
| hsa-mir-4666b | MI0019299 | chrX | 29574278 | 29574358 | + | - |
| hsa-mir-6086 | MI0020363 | chrX | 13590292 | 13590346 | + | - |
| hsa-mir-6089-1 | MI0020366 | chrX | 2609191 | 2609254 | + | - |
| hsa-mir-6134 | MI0021279 | chrX | 28495555 | 28495663 | - | - |
| hsa-mir-6857 | MI0022703 | chrX | 53405673 | 53405765 | - | - |
| hsa-mir-6858 | MI0022704 | chrX | 154450320 | 154450386 | + | - |
| hsa-mir-6894 | MI0022741 | chrX | 53198889 | 53198945 | - | - |
| hsa-mir-6895 | MI0022742 | chrX | 53195411 | 53195488 | - | - |
| hsa-mir-8088 | MI0025924 | chrX | 52336557 | 52336642 | - | - |
| hsa-mir-12129 | MI0039731 | chrX | 153335462 | 153335537 | + | - |

**Supplemental table 2**: DIANA-mirPath pathway analysis performed on X-resident miRNAs

| **KEGG pathway** | **p-value** | **genes** | **miRNAs** |
| --- | --- | --- | --- |
| Proteoglycans in cancer | 1.75465067983e-13 | 132 | 61 |
| Viral carcinogenesis | 7.94267483036e-13 | 137 | 61 |
| Protein processing in endoplasmic reticulum | 8.20335310862e-13 | 123 | 63 |
| Cell cycle | 6.68352721051e-11 | 92 | 58 |
| Ubiquitin mediated proteolysis | 2.40559375266e-10 | 101 | 62 |
| Adherens junction | 3.79135962402e-10 | 58 | 55 |
| Endocytosis | 6.13174513928e-10 | 138 | 62 |
| Fatty acid metabolism | 1.26279897769e-09 | 27 | 38 |
| Bacterial invasion of epithelial cells | 1.8911373008e-09 | 59 | 53 |
| Renal cell carcinoma | 6.68966608765e-09 | 51 | 49 |
| Spliceosome | 5.24542954393e-08 | 91 | 57 |
| Hippo signaling pathway | 7.03879516222e-07 | 89 | 56 |
| Fatty acid biosynthesis | 7.04847255397e-07 | 6 | 18 |
| TGF-beta signaling pathway | 7.46862185658e-07 | 53 | 51 |
| Chronic myeloid leukemia | 1.6332852093e-06 | 54 | 57 |
| Pathways in cancer | 1.91937678098e-06 | 233 | 62 |
| N-Glycan biosynthesis | 2.62295074532e-06 | 34 | 38 |
| Lysine degradation | 2.64971979058e-06 | 35 | 45 |
| Prostate cancer | 2.82370109996e-06 | 66 | 56 |
| Colorectal cancer | 4.61169973062e-06 | 47 | 49 |
| Shigellosis | 4.61169973062e-06 | 47 | 50 |
| Hepatitis B | 4.61169973062e-06 | 90 | 58 |
| p53 signaling pathway | 9.45540364374e-06 | 51 | 52 |
| Glioma | 1.57760090654e-05 | 44 | 53 |
| Regulation of actin cytoskeleton | 2.77970806677e-05 | 127 | 58 |
| Neurotrophin signaling pathway | 3.47267066317e-05 | 80 | 56 |
| Pancreatic cancer | 3.84575433301e-05 | 49 | 49 |
| Prion diseases | 4.08694505622e-05 | 16 | 33 |
| Thyroid cancer | 4.10944945962e-05 | 23 | 42 |
| Oocyte meiosis | 4.38220862593e-05 | 68 | 54 |
| Focal adhesion | 4.57856258171e-05 | 128 | 60 |
| Arrhythmogenic right ventricular cardiomyopathy (ARVC) | 5.0490163565e-05 | 42 | 49 |
| Endometrial cancer | 8.91195728161e-05 | 38 | 51 |
| FoxO signaling pathway | 0.00010247 | 87 | 61 |
| Acute myeloid leukemia | 0.00033645 | 41 | 46 |
| Bladder cancer | 0.00039893 | 31 | 49 |
| Central carbon metabolism in cancer | 0.00050909 | 44 | 53 |
| Non-small cell lung cancer | 0.00091851 | 39 | 50 |
| Insulin signaling pathway | 0.00109445 | 87 | 56 |
| HTLV-I infection | 0.00109445 | 151 | 62 |
| mRNA surveillance pathway | 0.00120836 | 62 | 50 |
| Epstein-Barr virus infection | 0.00156971 | 120 | 61 |
| mTOR signaling pathway | 0.00315918 | 41 | 45 |
| Steroid biosynthesis | 0.00318261 | 13 | 26 |
| HIF-1 signaling pathway | 0.00318261 | 66 | 57 |
| Wnt signaling pathway | 0.00318261 | 83 | 59 |
| ErbB signaling pathway | 0.00456278 | 53 | 55 |
| Small cell lung cancer | 0.00588598 | 54 | 54 |
| MAPK signaling pathway | 0.00602682 | 141 | 59 |
| Glycosaminoglycan biosynthesis - keratan sulfate | 0.00699432 | 9 | 13 |
| Estrogen signaling pathway | 0.01065524 | 57 | 52 |
| RNA transport | 0.01084067 | 96 | 59 |
| Base excision repair | 0.01088986 | 20 | 28 |
| Biotin metabolism | 0.0144803 | 2 | 3 |
| Progesterone-mediated oocyte maturation | 0.01569078 | 54 | 49 |
| Fc gamma R-mediated phagocytosis | 0.01582362 | 55 | 52 |
| Lysosome | 0.01582362 | 69 | 55 |
| Notch signaling pathway | 0.01588246 | 32 | 33 |
| DNA replication | 0.01849486 | 24 | 27 |
| TNF signaling pathway | 0.01849486 | 66 | 55 |
| Thyroid hormone signaling pathway | 0.01982655 | 69 | 58 |
| AMPK signaling pathway | 0.02146775 | 74 | 55 |
| Sphingolipid signaling pathway | 0.02241488 | 68 | 49 |
| Transcriptional misregulation in cancer | 0.02241488 | 101 | 59 |
| Circadian rhythm | 0.03357502 | 21 | 40 |
| Vitamin B6 metabolism | 0.03432853 | 5 | 10 |
| Fatty acid elongation | 0.03706275 | 11 | 20 |
| Prolactin signaling pathway | 0.03706275 | 44 | 53 |
| Glycosaminoglycan biosynthesis - chondroitin sulfate / dermatan sulfate | 0.04323062 | 12 | 23 |
| Melanoma | 0.04569329 | 41 | 53 |
| Biosynthesis of unsaturated fatty acids | 0.04774866 | 13 | 27 |
| Other types of O-glycan biosynthesis | 0.04774866 | 17 | 29 |
| Signaling pathways regulating pluripotency of stem cells | 0.04842049 | 78 | 59 |
| RNA degradation | 0.04856323 | 47 | 49 |
